# Nicotinamide N-Methyltransferase in Acquisition of Stem Cell Properties and Therapy Resistance in Cancer

**DOI:** 10.3390/ijms22115681

**Published:** 2021-05-26

**Authors:** Renata Novak Kujundžić, Marin Prpić, Nikola Đaković, Nina Dabelić, Marko Tomljanović, Anamarija Mojzeš, Ana Fröbe, Koraljka Gall Trošelj

**Affiliations:** 1 Laboratory for Epigenomics, Division of Molecular Medicine, Ruđer Bošković Institute, 10000 Zagreb, Croatia; Marko.Tomljanovic@irb.hr (M.T.); Anamarija.Mojzes@irb.hr (A.M.); troselj@irb.hr (K.G.T.); 2 Department of Oncology and Nuclear Medicine, University Hospital Center Sestre Milosrdnice, 10000 Zagreb, Croatia; marin.prpic@kbcsm.hr (M.P.); nina.dabelic@kbcsm.hr (N.D.); afrobe@irb.hr (A.F.); 3 Department of Clinical Oncology, School of Medicine, University of Zagreb, 10000 Zagreb, Croatia; nikola.dakovic@kbcsm.hr; 4 School of Dental Medicine, University of Zagreb, 10000 Zagreb, Croatia; 5 Institute for Clinical Medical Research and Education, University Hospital Center Sestre Milosrdnice, 10000 Zagreb, Croatia

**Keywords:** nicotinamide N-methyltransferase, nicotinamide adenine dinucleotide, aldehyde oxidase, cancer, epigenetic reprogramming, senescence, stem cell properties, therapy resistance

## Abstract

The activity of nicotinamide N-methyltransferase (NNMT) is tightly linked to the maintenance of the nicotinamide adenine dinucleotide (NAD^+^) level. This enzyme catalyzes methylation of nicotinamide (NAM) into methyl nicotinamide (MNAM), which is either excreted or further metabolized to N1-methyl-2-pyridone-5-carboxamide (2-PY) and H_2_O_2_. Enzymatic activity of NNMT is important for the prevention of NAM-mediated inhibition of NAD^+^-consuming enzymes poly–adenosine -diphosphate (ADP), ribose polymerases (PARPs), and sirtuins (SIRTs). Inappropriately high expression and activity of NNMT, commonly present in various types of cancer, has the potential to disrupt NAD^+^ homeostasis and cellular methylation potential. Largely overlooked, in the context of cancer, is the inhibitory effect of 2-PY on PARP-1 activity, which abrogates NNMT’s positive effect on cellular NAD^+^ flux by stalling liberation of NAM and reducing NAD^+^ synthesis in the salvage pathway. This review describes, and discusses, the mechanisms by which NNMT promotes NAD^+^ depletion and epigenetic reprogramming, leading to the development of metabolic plasticity, evasion of a major tumor suppressive process of cellular senescence, and acquisition of stem cell properties. All these phenomena are related to therapy resistance and worse clinical outcomes.

## 1. Introduction

Acquired resistance to anti-cancer agents is one of the major obstacles to the successful treatment of cancer patients [1,2]. Despite major developments in treatment strategies, most disseminated cancers still remain fatal [3]. Programs of cellular senescence, and acquisition of stem cell properties, are tightly interwoven during the development of therapy resistance [4,5]. In recent years, it is becoming evident that cancer-related changes in metabolism, in addition to being necessary for uncontrolled proliferation of cancer cells, profoundly influence cellular epigenetic make-up and regulate cellular programs central to cancer cell aggressiveness [6]. Nicotinamide adenine dinucleotide (NAD^+^), and its metabolites (nicotinamide, 1-methylnicotinamide, and N1-methyl-2-pyridone-5-carboxamide), have central roles in metabolism-induced epigenetic regulation, enabling cells to adapt to various types of stress. The NAD^+^-associated adaptive responses relate to its cofactor role in numerous oxidation-reduction reactions related to energy production, in which NAD^+^ serves as the universal electron transporter without being consumed [7,8,9]. On the other hand, NAD^+^-consuming reactions are dependent on regulatory enzymes, such as sirtuins, ADP-ribosyltransferases, and cyclic ADP-ribose cyclases. These enzymes consume NAD^+^ by cleaving it to nicotinamide (NAM) and acetyl adenosinediphosphate ribose (AADPR), adenosinediphosphate ribose (ADPR), or cyclic ADPR (cADPR), respectively [10,11,12]. These NAD^+^-consuming signaling reactions participate in regulation of virtually all cellular functions by poly- and mono-ADP ribosylation, sirtuin-mediated protein deacetylation, and the generation of calcium-mobilizing cADPR [13,14,15,16,17]. Accordingly, NAD^+^ is important in many signaling reactions, including regulation of the cell cycle, DNA repair, transcription, and calcium signaling.

Imbalance between NAD^+^ consumption and biosynthesis has a profound impact on cellular physiology and drives multiple pathologies, including cancer. Considering the high rate of NAD^+^ consumption in signaling reactions, its re-synthesis from NAM in salvage pathway is of vital importance [18,19]. In this pathway, NAM phosphoribosyltransferase (NAMPT) converts NAM into the nicotinamide mononucleotide (NMN), which nicotinamide mononucleotide adenylyl-transferases 1–3 (NMNATs 1–3) convert into NAD^+^ [10]. High affinity of NAMPT to NAM allows for a very efficient recycling of NAM into NAD^+^ [20].

**Figure 1:**
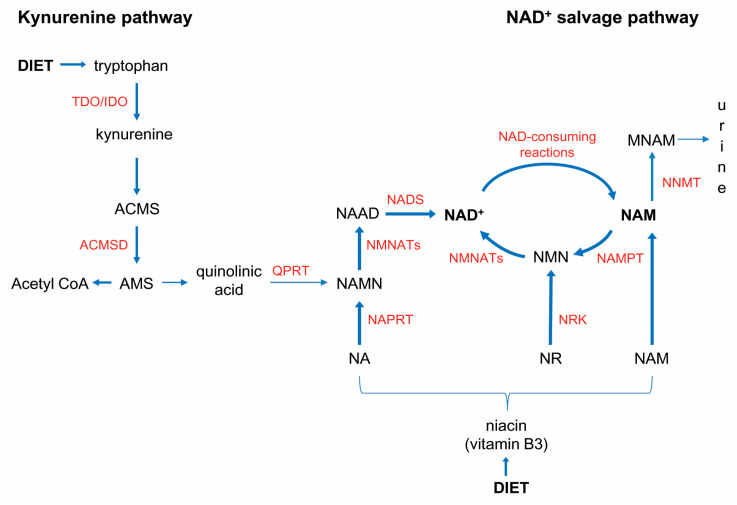
NAD^+^ biosynthesis pathways. Human dependency on vitamin B3, for NAD^+^ synthesis, is based on high activity of alpha-amino-beta-carboxy-muconatesemialdehyde decarboxylase (ACMSD), which diverts alpha-amino-beta-carboxy-muconatesemialdehyde (ACMS) away from NAD^+^ synthesis. Nicotinamide (NAM) is the main precursor for NAD^+^ synthesis and the byproduct of NAD^+^-consuming enzymes. Under physiological conditions, de novo synthesis of NAD^+^ from tryptophan, in the kynurenine pathway, takes place mainly in the liver. The main dietary precursors for NAD^+^ synthesis are nicotinic acid (NA), nicotinamide riboside (NR) and nicotinamide (NAM), collectively termed vitamin B3 or niacin. Endogenously, NAM is generated as a byproduct of NAD^+^-consuming reactions. Under physiological conditions, most of the NAM generated in NAD^+^-consuming reactions is recycled to NAD^+^. Nicotinamide N-methyltransferase (NNMT) is a safeguard, which in conditions of either overwhelming NAM generation in NAD^+^-consuming reactions, or dietary surplus, methylates NAM to prevent NAM’s inhibitory effect on PARPs and SIRTs.

Alternatively, NAD^+^ can be *de novo* synthesized from tryptophan (Trp) in kynurenine pathway. This metabolic pathway is, in humans, less efficient than in mice. The reason for this is high activity of alpha-amino-beta-carboxy-muconatesemialdehyde decarboxylase (ACMSD), an enzyme which diverts alpha-amino-beta-carboxy-muconatesemialdehyde (ACMS), an intermediate metabolite in the kynurenine pathway, away from NAD^+^ synthesis by limiting its spontaneous cyclization into quinolinic acid (Figure 1) [21]. For this reason, humans utilize ~60–70 mg Trp to generate an equivalent amount of NAD^+^ produced from 1 mg niacin, the dietary precursors of NAD^+^, when fed an experimental, Trp-rich, and niacin-free, diet [22]. Niacin, or vitamin B3, is defined, collectively, as nicotinic acid, NAM, and nicotinamide riboside.

Deregulated NAD^+^ metabolism is a hallmark of many age-related diseases, including cancer [19,23,24,25]. In response to diverse cellular stresses, both NAD^+^ level, and the adaptability of NAD^+^ metabolism, underlies epigenetically-regulated metabolic reprogramming, commonly leading to the acquisition of phenotypic plasticity and therapy resistance [8,26,27,28]. The enzyme nicotinamide N-methyltransferase (NNMT), which transfers methyl units from S-adenosyl methionine (SAM) to NAM, has emerged as an important contributor to those processes [24].

Here, we review the role of NNMT in cancer, with respect to its involvement in dynamic process of transition from tumor-suppressive state of cellular senescence to stem cell-like phenotypic state, related to the acquisition of therapy resistance.

## 2. Cellular Senescence, Circumventing Senescence, and the Onset of Cancer Therapy Resistance

Malignant tumors are composed of heterogeneous populations of both neoplastic and stromal cells. Phenotypic and functional heterogeneity is a result of genetic and epigenetic alterations which, together with microenvironmental cues, strongly contribute to dynamic, and reversible, modifications of cellular properties [29,30]. After an initial period of response, tumor cells’ heterogeneity and plasticity confer great adaptability to therapy, leading to resistance. It is increasingly recognized that both conventional cytostatic, and targeted, therapies may promote the emergence of resistant cells, with cancer stem cell properties, through clonal selection of cell populations harboring genetic mutations and specific epigenetic alterations [31,32,33,34,35,36]. Cellular heterogeneity and plasticity are not inherent only to cancer cells but also, to other cellular components of a tumor microenvironment (TME). Stromal compartment has the capacity to promote tumor cell plasticity and proliferation by providing metabolic, and epigenetic, cues [28,37,38,39]. The onset of senescence facilitates the acquisition of stem cell properties [40].

### 2.1. Dual Roles of Cellular Senescence in Cancer

From the earliest stages in cancer development, cells are exposed to various types of stress and are under pressure to evolve strategies to counteract stress in order to survive. Cellular senescence is an important failsafe mechanism aimed to minimize damage, and preserve integrity, by instructing damaged, potentially dangerous, cells to cease dividing. In the context of cancer, senescence can be a double-edged sword. Although being tumor-suppressive in a cell-autonomous setting, cellular senescence of stromal cells promotes tumorigenesis [41]. These highly metabolically active cells, commonly referred to as having a senescence-associated secretory phenotype (SASP), secrete numerous growth factors, pro-inflammatory cytokines, and factors that change extracellular matrix, making it permissive for cancer progression [42,43,44].

Cellular senescence occurs in response to most forms of anti-cancer treatments [45,46]. Although therapy-induced senescence (TIS) is a desirable effect, in terms of halting tumor cell proliferation, SASP can induce or reinforce senescence, in an autocrine or paracrine manner, thereby promoting inflammation, occurrence of secondary tumors, and cancer relapse [47].

Chemotherapy-induced senescence has been recognized as an adaptive mechanism, which promotes the development of cancer cell plasticity and therapy resistance [48]. Moreover, senescence-like cell cycle arrest may not be a permanent state. Rather, it is a reversible condition. Under the sustained influence of pro-tumorigenic cytokines, cells can circumvent senescence, resume proliferation, and acquire stem cell properties [5].

### 2.2. Acquisition of Stem Cell Properties in Cancer

Acquisition of stem cell properties, in cancer cells, may have detrimental effects regarding cancer aggressiveness and resistance to therapy. The process in which non-motile, epithelial cells change their phenotype to become mesenchymal cells, endowed with invasive capacities, is termed epithelial to mesenchymal transition (EMT) [49]. Physiologically, EMT occurs during embryogenesis to enable cells to migrate in order to form new tissues and organs, and it has a central role in the wound healing process [50,51]. In cancer, EMT allows malignant, but still differentiated, cells to lose their epithelial characteristics and become migratory, able to invade tissues around the primary tumor, extravasate into lymphatics or blood vessels, and migrate to distant sites in the body [52]. Forced expression of EMT-inducing transcription factors (EMT-TFs), or treatment with transforming growth factor-β (TGFβ), activates EMT in normal and neoplastic epithelial tissues, resulting in the occurrence of cells with stem cell-like properties. In cancer, those cells are often referred to as cancer stem cells (CSCs). They make a minor cell population within a tumor, with high expression of cell surface marker CD44 and low expression of CD24, which distinguishes them from their non-CSC counterparts [53,54]. Cancer stem cells have the ability to self-renew and to differentiate into cells that form the bulk of the tumor. Similarly, non-CSCs can de-differentiate, implying bidirectional inter-conversion between cells with stem cell properties and differentiated cells [55]. Compared to their differentiated counterparts, CSCs are more resistant to anti-cancer therapies, resulting in their persistence after finalization of the therapy and leading to tumor relapse [56,57].

### 2.3. Interwoven Processes of Senescence and EMT—A Major Culprit in Therapy Resistance

**Figure 2:**
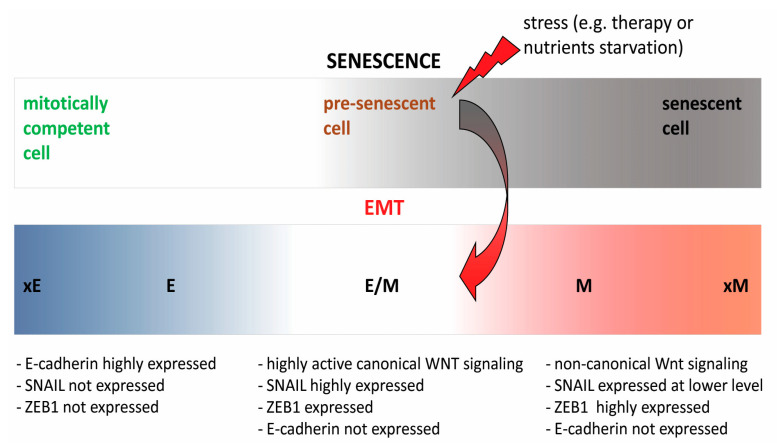
Schematic representation of different stages in the spectrum of cellular senescence and EMT. Circumventing senescence, precipitated by stress (e.g., therapy or nutrients starvation), accompanied by the acquisition of stem cell properties gives rise to highly proliferative, tumorigenic cells, unable to proceed either to fully senescent (mitotically incompetent) or fully mesenchymal state (xE and E-epithelial; E/M-hybrid epithelial/mesenchymal; M and xM-mesenchymal).

Much like senescence-associated cell cycle arrest is not an irreversible, permanent, state, oncogenic EMT is a transient, reversible process, followed by mesenchymal to epithelial transition (MET), to allow the proliferation of disseminated cancer cells at distant sites [58,59,60]. Considering that both senescence and EMT programs proceed through a whole spectrum of events, from starting point (a mitotically competent cell, in the case of senescence, and a fully epithelial cell, in the case of EMT) to end point (a completely senescent or fully mesenchymal cell) (Figure 2), the most important question is: What interfering events govern the escape of cancer cells from completing senescence or EMT programs, neither of which would allow fatal progression of cancer?

Programs of cellular senescence and EMT seem to be co-regulated by overlapping signaling networks. Signaling molecules, like TP53, CDKN2A (P16^INK4A^), and histone methyltransferase SUV39H1, are involved in the execution of the senescence program. These molecules have a critical role in preventing premature stem cells exhaustion and represent a barrier to conversion of normal cells into induced pluripotent cells [61,62].

Therapy-induced senescence (TIS)-associated stemness of lymphoma cells is related to enriched canonical Wnt signaling pathway [4]. Canonical Wnt signaling is also highly active in cells residing in a hybrid epithelial/mesenchymal (E/M) phenotypic state. Cancer cells, with hybrid E/M phenotype, are highly invasive, have strong tumor-initiating ability and metastatic potential. Persistence of such intermediate, hybrid phenotypic state depends on an incomplete execution of EMT program to fully mesenchymal state. Achievement of highly mesenchymal state, accompanied by significant loss of tumorigenicity, is dependent on the expression of transcription factor ZEB1 and the switch from canonical to non-canonical Wnt signaling [63]. The importance of canonical Wnt signaling both in highly tumorigenic E/M state and in acquisition of stem cell characteristics in TIS lymphoma cells further strongly points to the functional interplay between senescence- and stemness-controlling signaling networks.

Upon treatment with doxorubicin (adriamycin), the activation of canonical Wnt signaling has been observed in TIS-capable human cells (Suv39h1 expressing), but not in TIS-incapable SUV39H1^−^; BCL2 lymphomas. In TIS lymphoma cells, activated MEK–MAPK and PI3K–Akt signaling inhibits β-catenin degradation-promoting glycogen synthase kinase 3β (GSK3β) and serves as a cell-autonomous driver of canonical Wnt signaling, independent of Wnt ligand-receptor stimulation [4].

Circumvention of oncogene-induced cellular senescence, in pancreatic ductal adenocarcinoma cells, has been shown to be accompanied by re-activation of signal transducer and activator of transcription 3 (STAT3), an increased reliance on mitochondrial functions and acquisition of stem cell properties [5]. Decreased cellular NAD^+^ level has been documented to activate STAT3 and promote EMT in human lung cancer cell line A549 and human liver cancer cell line HepG2. The decrease in cellular NAD^+^ level in the cited study was achieved through overexpressing CD38, a NAD^+^ hydrolase, or by FK866, pharmacological inhibitor of NAMPT. In CD38 overexpressing cells, diminished NAD^+^ level was accompanied by an increase in NNMT expression [64,65]. It has, recently, been reported that during aging and in response to inflammation, concomitant with accumulation of pro-inflammatory M1 macrophages expressing high level of CD38, NAD^+^ level declines. Pro-inflammatory cytokines, secreted by senescent cells (characteristic of SASP), induce macrophages to proliferate and express CD38, thereby lowering NAD^+^ level [66].

Considering that senescent cells facilitate the onset of EMT, through their negative influence on the NAD^+^ level, understanding of molecular mechanisms that orchestrate NAD^+^-dependent fine balance between, generally considered, tumor-suppressive process of cellular senescence and its reversion into pro-tumorigenic EMT program is of highest importance.

## 3. The Role of NNMT in Acquisition of Stem Cell Properties and Therapy Resistance

The renewed scientific interest in tumorigenesis-related metabolic reprogramming, supported by the developments in research technology, led to many high throughput genomic, proteomic, and metabolomic studies, which have uncovered many metabolic enzymes with altered expression/activity in cancers. Metabolism is closely linked to epigenetic changes and post-translational modifications of proteins, thereby having a profound influence on signaling and transcriptional networks. Molecular mechanisms, underlying their function in cancer, are still not completely understood.

One of the metabolic enzymes, NNMT, is often over-expressed in various types of cancer, where it regulates NAD^+^ consumption-recycling flux and cellular methylation potential [19,67,68].

### 3.1. NNMT

NNMT (E.C.2.1.1.1) is the enzyme which methylates nicotinamide (NAM), using the universal methyl donor S-adenosyl methionine (SAM), to produce 1-methylnicotinamide (MNAM) and S-adenosyl-L-homocysteine (SAH). MNAM can be excreted from the body directly, or, before excretion, oxidized by aldehyde oxidase 1 (AOX1) into N1-methyl-2-pyridone-5-carboxamide (2-PY) and N1-methyl-4-pyridone-3-carboxamide (4-PY) [69].

The human *NNMT* gene (Gene ID: 4837), located on chromosome 11, is composed of three exons and two introns. It produces canonical mRNA of 1578 nucleotides coding for a protein of 264 amino acids. Splice variants exist, they represent a very small fraction of total *NNMT* transcripts, and their functional significance is currently unexplored [69].

Transcription factors STAT3 and hepatocyte nuclear factor 1 β (HNF1 β) have been documented to increase *NNMT* promoter activity in cancer cells [70,71], but, beyond that, its transcriptional regulation has, for long time, remained rather unexplored. It has recently been reported that BRCA1 DNA repair associated (BRCA1) deficiency, in ovarian cancer cells, upregulates NNMT expression, as BRCA1 binding to a *NNMT* promoter negatively regulates *NNMT* transcription [72]. Considering that NNMT expression is elevated under conditions favoring reactive oxygen species (ROS) production and increased mitochondrial activity, the question is whether the *NNMT* promoter is regulated by transcription factors involved in cellular antioxidant response, such as NF-E2-Related Factors 1 and 2 (NRF1 and NRF2). To the best of our knowledge, there is only one report on the relationship between Nrf1 and *Nnmt* expression. In the liver of Nrf1-conditional knockout mice, the expression of *Nnmt* is significantly down-regulated [73].

The expression levels of *NNMT* transcripts and proteins differ considerably in several organs (pancreas, muscles, kidney, gastrointestinal tract), implying complex regulation of expression in a tissue-specific manner [74]. Under physiological conditions, NNMT is most abundantly expressed in the liver, with lower levels found in adipose tissue, muscle, and fibroblasts. A very low level of NNMT is found in the central nervous system and hematopoietic cells [69]. NNMT has an important role in regulating efficient recycling of NAM into NAD^+^.

Bockwoldt et al. recently elaborated that the significance of evolutionary emergence of NNMT, in vertebrates, is prevention of NAM-mediated inhibition of NAD^+^-consuming signaling reactions by facilitating its excretion. Uninhibited NAD^+^-consuming reactions generate NAM to be recycled back to NAD^+^ in the salvage pathway, preserving functionality of this main pathway of NAD^+^ synthesis in humans. Different affinity of NAMPT, and NNMT, for NAM (*K_M_* of 5 nM and *K_M_* 400 μM, respectively) suggests that only high increase in NAM level would activate NNMT to prevent NAM accumulation and inhibition of NAD^+^-consuming reactions. Calculations of steady-state NAD^+^ concentrations and NAD^+^ consumption flux, by simulating NAD^+^ biosynthesis via NAMPT, in the presence or absence of NNMT, predicted that the presence of NNMT enables higher, rather than lower, NAD^+^ consumption fluxes, although diminishing the steady-state concentration of NAD^+^. Most importantly, Bockwoldt et al. further showed that, when NAD^+^ concentration drastically drops, due to high expression of NNMT, NAD^+^ consumption declines [19].

### 3.2. NNMT-Mediated “Nicotinamide Drain” and NAD^+^ Depletion

**Figure 3:**
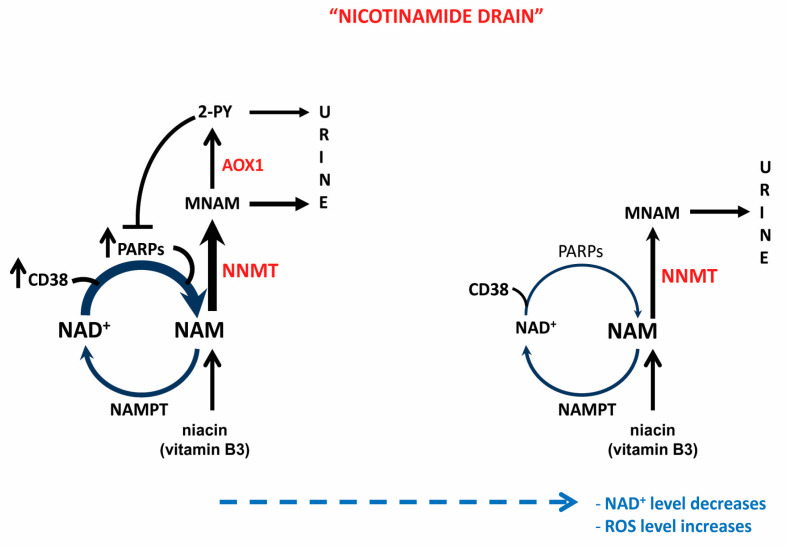
“Nicotinamide drain”—mediated NAD^+^ decrease. Overactivation of NAD^+^-consuming reactions causes extreme elevation of NAM in the cell, exceeding the capacity of the salvage pathway to recycle it into NAD^+^. The surplus of NAM is methylated by NNMT into MNAM to facilitate its excretion and prevent inhibition of NAD^+^-consuming reactions, thereby preserving cellular NAD^+^ flux. Part of generated MNAM is further metabolized by AOX1 to 2-PY, which, in addition to being excreted, inhibits PARPs, thereby limiting liberation of NAM for recycling into NAD^+^ and, over time, leading to depletion of cellular NAD^+^ level.

In vivo, uninhibited, but limited, NAD^+^ consumption flux (i.e., PARP activation) would generate NAM to be faithfully recycled into NAD^+^, in the absence of alternative inhibitors of NAD^+^-consuming reactions. Due to relatively low affinity of human NNMT for NAM (*K_M_* approximately, 400 μM), only the high increase in dietary NAM intake leads to an elevation in MNAM level under physiological conditions [67]. The increase in MNAM level has been, however, observed in various cancer cell lines and cancer types, in which NNMT expression was likewise elevated, but the source of NAM was not explored [67,75,76,77]. It is conceivable that high activity of NAD^+^-consuming reactions is responsible for an overwhelming liberation of NAM in cancer. To prevent NAM’s inhibitory effect on NAD-consuming reactions, NNMT would exclude it from the system by methylation. This could, depending on the extent by which NAM level exceeds the capacity of NAMPT to recycle it to NAD^+^, down-regulate the cellular NAD^+^ level. Largely overlooked, when considering the role of NNMT and its product MNAM, is the involvement of aldehyde oxidase (AOX1) in metabolizing MNAM into 2-PY, a strong PARP inhibitor [78]. Inhibition of PARP, by 2-PY, stalls regeneration of NAM needed for NAD^+^ synthesis. This creates a “NAM drain” that disrupts the balance between NAD^+^-consumption and synthesis, diminishing the NAD^+^ level (Figure 3).

### 3.3. NNMT and Pluripotency

Pluripotency in embryogenesis, much like the senescent state in senescence and mesenchymal state in EMT, does not represent a single defined state. Diverse states of pluripotency exist, with characteristic epigenetic and gene expression profiles directing cellular phenotype. Sperber et al. showed that NNMT has an important role in regulating pluripotency of human embryonic stem cells (hESC). When comparing hESCs in two states of pluripotency, preimplantation, naïve, and postimplantation, primed, they have observed highly upregulated NNMT and MNAM in naïve compared to primed hESCs. In naïve hESCs, NNMT is required for maintenance of low level of suppressive histone mark, H3K27me3, which is needed for repression of the Wnt pathway in primed hESCs. Knockdown of NNMT, in naïve hESCs, increased H3K27me3 repressive marks in developmental and key metabolic genes, responsible for the metabolic switch needed for transition from naïve to primed state. A significant H3K27me3 increase was observed in the promoter of metabolic gene *EGLN1*, and was well-correlated with its lower expression in primed hESCs. Downregulation of the corresponding protein, prolyl hydroxylase 2 (PHD), prevented hydroxylation and ubiquitination-dependent degradation of HIF1α. Stabilization of HIF1α in primed hESCs caused a metabolic switch from bivalent (equally competent for mitochondrial respiration and glycolysis), in naïve cells, to highly glycolytic in primed hESCs [79]. HIF1α knock-out prevented naïve to primed transition in hESCs [80]. Upon further differentiation, primed hESCs rapidly reprogram their metabolism to highly mitochondrial respiration. It has been suggested that a metabolic switch towards glycolysis (cancer-characteristic Warburg effect) in primed hESCs depends on the drop in NNMT activity [79].

Cellular metabolism and signaling are tightly connected, and regulate each other, to modulate stem cell functions, survival, and proliferation. Since distinct forms of NNMT-regulated histone methylations direct a metabolic switch between cells residing in different phenotypic states (naive to primed hESC; differentiated somatic cells vs. iPSCs), it is conceivable that metabolic reprogramming has a causative role, rather than being a mere consequence of acquisition of stem cell properties [81,82].

### 3.4. NNMT and Epigenomic Reprogramming in Cancer

Epigenomic reprogramming is the main consequence of NNMT overexpression/activity in cancer. NNMT indirectly, through depleting SAM, impairs methylation of histones and other proteins in cancer cells. Ulanovskaya et al. found that NNMT does not equally influence all of the histone methylation events, nor does it influence global levels of DNA methylation. They proposed that its selectivity for certain methylation reactions depends on relative *K_M_* value of individual methyltransferases for SAM. Methyltransferases with higher *K_M_* values for SAM (EZH1-SAM *K_M_* 2.5 µM; EZH2-SAM *K_M_* 1.2 µM; LCMT1-SAM *K_M_* 1.3 µM) tend to be more sensitive to NNMT activity. The effect of NNMT on SAM level was, however, observed only in cancer cells grown in media with low (10–20 μM), but not in high (100 μM), methionine concentration [67].

NNMT expression is elevated in many types of malignant tumors, but in liver cancer, its expression has been found to be reduced, although abundant, when compared to adjacent, non-transformed hepatic tissue. Regardless of overall reduced NNMT expression in liver cancer, within hepatocellular carcinoma samples, higher levels of NNMT positively correlated with tumor stage and shorter disease-free survival. The reduced expression of NNMT has been observed in stage II HCC, compared to stage I tumors. However, the expression level of NNMT in stage III and IV returned to the level observed in stage I tumors [83]. Considering that NNMT is a negative regulator of autophagy, its transient, reduced expression in liver cancer could promote cancer cells survival, under nutrient starvation, by activating autophagy [84]. It has been proposed that reduced expression of NNMT may precede tumor invasion. Subsequent increase in NNMT expression in later stages of disease progression may be due to tumor de-differentiation, preceding tumor invasion [83]. Accordingly, in hepatocellular carcinoma, NNMT downregulates H3K27 methylation and transcriptionally activates a cluster of differentiation 44 (CD44), a stem cell marker. Moreover, NNMT’s product MNAM stabilizes the CD44 protein by inhibiting its ubiquitin-mediated degradation [85].

In addition to down-regulating SAM level, and SAM/SAH ratio, in the course of its activity, NNMT participates in the regulation of methyl donor balance, independently of its enzymatic activity. It has been shown that mutant, inactive, NNMT interacts with essential enzymes in methionine cycle: methionine adenosyltransferase Iα (Mat1a) that forms SAM; adenosylhomocysteinase (Ahcy) which degrades S-adenosylhomocysteine (SAH) to adenosine and homocysteine; and betaine-homocysteine methyltransferase (Bhmt) which uses betaine and homocysteine to produce dimethylglycine and methionine. Interaction between NNMT and these enzymes implies the possibility that NNMT, regardless of its activity, promotes the recycling of methyl donor metabolites by bringing components of the methionine cycle into the proximity [86].

The exact mechanism by which NNMT exerts its effect on particular methylation reaction, beyond influencing cellular SAM level, warrants further investigation since histone methylation profoundly influences crucial physiological processes, such as embryogenesis, as well as pathological states.

In addition to being expressed in cancer cells, the presence of NNMT has been documented in metastasis-associated stroma of high grade ovarian serous carcinoma (HGSC). Rather than its expression in tumor cells, stromal expression of NNMT drives the development of cancer associated fibroblasts (CAF) phenotype, with characteristic expression of CAF markers, secretion of pro-oncogenic cytokines, and extracellular matrix-modifying factors. Expression of NNMT in CAFs, and consequential depletion of SAM, causes widespread changes in gene expression in the tumor stromal cells. Knockdown of *NNMT*, in CAFs, reversed their morphology to normal omental fibroblasts [87].

NNMT expression was associated with enriched expression of genes related to EMT. Moreover, the impact of NNMT expression in CAFs on tumor progression and metastasis was further supported by its overexpression in normal fibroblasts, which promoted cancer cell proliferation in vitro. Knockdown of NNMT in CAFs, on the contrary, attenuated cancer cell proliferation and chemotaxis. Upon NNMT knockdown, NAD^+^ levels increased [87].

By regulating cellular NAD^+^ and NAM level, NNMT expression and activity has a profound impact on NAD^+^-dependent deacetylation reactions, mediated by sirtuins, in a tissue specific manner. Overexpression of NNMT in white adipose tissue decreases NAD^+^ level, whereas, in liver, it does not have such an effect [87,88]. In addition to the effect of NNMT-mediated regulation of NAD^+^ and NAM level on sirtuin’s activity, NNMT, through MNAM, stabilizes SIRT1 protein by preventing its polyubiquitylation and degradation in proteasome. The precise mechanism by which MNAM inhibits SIRT1 polyubiquitylation has not been investigated [89]. However, it has been proposed that either MNAM or its metabolites inhibit ubiquitin ligase [90].

An increased expression of NNMT, in prostate cancer cells PC-3, stabilizes SIRT1 and contributes to cellular migratory potential and invasiveness, which can be overcome by exposure to an elevated level of SIRT1 inhibitor-NAM [91]. In breast cancer patients, NNMT expression was reported to correlate with shorter survival and resistance to chemotherapy.

Overexpression of NNMT in breast cancer cell lines SK-BR-3 (ER−, HER2+) and MCF7 (ER+, HER2−), which constitutively do not express NNMT, significantly inhibited doxorubicin (adriamycin)- and paclitaxel-mediated apoptotic cell death and suppression of colony formation. In line with this discovery, silencing of NNMT in MDA-MB-231 (ER−, HER2−) cell line, with high constitutive NNMT expression, enhanced their sensitivity to chemotherapy. It has been demonstrated that NNMT overexpression, while not increasing *SIRT1* transcripts, increases SIRT1 protein levels in SK-BR-3 and MCF7 cells. Inhibition of SIRT1 by EX527, or silencing of *SIRT1* by siRNA, abolished negative effects of NNMT on sensitivity to doxorubicin and paclitaxel. Results of this study suggest a critical role of SIRT1 in mediating NNMT-related therapy resistance in breast cancer [92].

Enzymes SIRT1 and PARP-1 negatively regulate each other through competition for common co-substrate NAD^+^ and by modifying each other, which regulates their catalytic activity [93]. The NNMT metabolite, 2-PY, has a strong inhibitory effect on PARP-1 [94]. Whether 2-PY has a role in upregulation of SIRT-1 activity deserves to be investigated in the context of NNMT-mediated SIRT1 stabilization. It could be consequential for the observed SIRT1 overexpression in HCC, where it promotes tumor invasiveness and migration by inducing EMT [95]. Considering that NNMT upregulation in the liver does not decrease NAD^+^ level, in contrast to adipose tissue, concomitant stabilization of SIRT1 by NNMT and the ability of liver to synthesize NAD^+^ *de novo* from Trp may allow for SIRT1 overactivation in this type of cancer. Further research is needed for understanding the precise mechanism/s, by which NNMT regulates SIRT1 activity, to appreciate their involvement in metabolic and phenotypic plasticity in a cell/tissue-specific context.

### 3.5. The Role of NNMT in Acquisition of Metabolic Plasticity and Stem Cell Properties in Cancer

A report on NNMT’s central role in the acquisition of metabolic plasticity and stem cell properties in ovarian serous carcinoma cells, exposed to chronic glucose deprivation, is in line with the role of NNMT in hESCs. The study was undertaken to examine the consequences of glucose restriction in high grade serous ovarian cancer (HGSC), which often has underdeveloped vasculature or is treated with anti-angiogenic agents, such as bevacizumab (recently FDA-approved for recurrent HGSC). When forced to adapt to nutritional stress, cancer cells reprogram their metabolism in order to survive. Cells cultured for a prolonged period (8 months), in low glucose media (~0.69 mM), were selected for metabolically plastic, glucose-independent cells. Contrary to respective parental cells, glucose restriction-resistant cells maintained their proliferation and colony-forming abilities in a low glucose medium. In all glucose-restricted cell lines, NNMT was up-regulated and has been demonstrated to be indispensable for their resistance to glucose deprivation. In addition to NNMT up-regulation, those cells acquired stem cell characteristics, as judged from the expression of stem cell specific transcription factor ZEB1, loss of E-cadherin, and up-regulation of mesenchymal markers vimentin and N-cadherin. NNMT was present in all examined cell lines with up-regulated ZEB1, but not all NNMT-positive cells were ZEB1-positive. Induced ZEB1 over-expression, in the parental NNMT-negative OVCAR3 cell line, resulted with robust induction of NNMT. Together, these data suggest existence of regulatory mechanisms able to govern ZEB1-independent acquisition, of stem cell properties, in NNMT positive cells [96].

The expression of ZEB1 is under control of several micro RNAs (for instance, miR-200c [97] and miR-130b [98] are known repressors of ZEB1). miR-130b is negatively regulated by TP53. The possible explanation for the lack of ZEB1 expression in Caov-3 cells, with high level of NNMT [96], may be the consequence of TP53 nonsense point mutation [99], which precludes TP53 suppressive effect on miR-130b and may be the reason for ZEB1 silencing.

Metabolic reprogramming, associated with glucose deprivation and NNMT expression, favors oxidative phosphorylation (OXPHOS) with elevated levels of ROS [96]. In *C. elegans*, lifespan extension, due to Sirt1 activation (a known consequence of fasting [100]), has been demonstrated to depend not only on the expression of anmt-1, a *C. elegans* NNMT orthologue and generation of MNAM, but on the expression of aldehyde oxidase GAD3. GAD3 is an orthologue of previously mentioned AOX1. In the process of forming two metabolites, 2-PY and 4-PY, AOX1 produces hydrogen peroxide. The effect of Sirt1 activity on *C. elegans* lifespan extension has been demonstrated to depend on both MNAM and GAD3 expression-mediated upregulation of ROS. In concentrations equimolar to the lifespan-extending concentration of MNAM, 2-PY, and 4-PY did not extend *C. elegans* lifespan [101]. In human cells, the effect of these two metabolites, and the consequences of an AOX1-mediated increase in ROS, warrants further investigation.

## 4. ROS and EMT

Moderate levels of ROS, accompanied with adequate antioxidant response, is beneficial for a healthy organism. Calorie restriction-associated moderate elevation of ROS, does not only extend lifespan of lower organisms, like *C. elegans*, but also boosts antioxidant defense in higher organisms, including humans. However, excessive production of ROS can cause DNA damage and genomic instability, leading to malignant transformation of cells and tumor progression [102].

Elevated levels of ROS alter gene expression [103] and stimulate cell invasiveness [104].

Prolonged (7 days) exposure of mouse mammary epithelial cells SCp2, to increased ROS levels (H_2_O_2_), induces EMT and the up-regulation of transcription factor Snail [102]. Snail mediates EMT by down-regulating expression of the gene coding for cell adhesion molecule E-cadherin (*CDH1*), through binding to several E-boxes in *CDH1* promoter [105].

Another facet of EMT-promoting effect of Snail is its involvement in regulation of alternative splicing. Derailed alternative splicing in cancer is associated with expression of a numerous cancer-related protein isoforms [106]. Snail binds E-boxes in the promoter of the epithelial splicing regulatory protein 1 (ESRP1), down-regulates its expression, and promotes CD44 isoform switching that is required for EMT [107].

CD44 is a transmembrane glycoprotein involved in various cellular functions. High expression of certain CD44 isoforms in cancer has been implicated in the acquisition of stem cell properties, metastasis, and therapy resistance [108]. The *CD44* gene consists of nine variable exons and nine constitutive exons. Alternative splicing of *CD44* mRNA results in two groups of transcript variants and corresponding protein isoforms: CD44v, which contain diverse combinations of variable exons and CD44s (CD44 standard), which lacks all variable exons. Gradual switch from CD44v (predominantly expressed in epithelial cells) to CD44s (expressed in mesenchymal cells) occurs in the course of EMT [107].

Elevation of ROS, in addition to inducing EMT, leads to the acquisition of cancer therapy resistance [109]. Recently, Miyazaki et al. reported a switch from CD44v to CD44s expression in head and neck carcinoma cells (HNCC) during the acquisition of cisplatin resistance. The underlying mechanisms were highly dependent on CD44s, which was shown to down-regulate miR-200c, a negative regulator of ZEB1. Thereby, ZEB1, a transcriptional repressor of E-cadherin, is up-regulated and relevant for induced EMT in cisplatin-resistant cells [110].

In addition to the involvement of ROS in EMT induction, through up-regulation of Snail, the forced overexpression of Snail leads to elevation of ROS. This phenomenon was shown by Barnett et al. in a model of prostate cancer cell line ARCaP. The expression analysis of oxidative stress-related genes, by Real Time PCR Array, revealed that Snail significantly increases expression of AOX1 (14.56 fold). Since AOX1 generates H_2_O_2_ in the course of its activity, the authors proposed upregulation of AOX1 as the most probable reason for ROS increase [111].

### 4.1. Aldehyde Oxidase (AOX1)

Aldehyde oxidase (AOX; EC1.2.3.1) is cytosolic enzyme that catalyzes oxidation of various xenobiotics and is the only enzyme that oxidizes NNMT’s product MNAM. Transcription of *AOX1* is regulated by NRF2 [112,113]. There is only one isoform of this enzyme in humans (AOX1), whereas four isoforms are present in mice (AOX1–4). Human AOX1 is a homodimer formed from two subunits of 150 kDa. To become catalytically active, AOX1 requires sulfuration of molybdenium cofactor MTC, which is accomplished by molybdenium cofactor sulfurase (MOCOS). In the course of its activity, AOX1 generates hydrogen peroxide. Potent inhibitor of AOX1 is raloxifene [114], a selective estrogen receptor modulator. When exploring inhibitory effects of different compounds on AOX1, using MNAM as a substrate, raloxifene was the most potent, followed by hydralazine (antihypertensive) and clozapine (antipsychotic). In a regular clinical therapeutic protocol, raloxifene and clozapine concentration, in plasma, is insufficient to interfere with MNAM metabolism through inhibition of AOX1. On the other hand, hydralazine inhibits AOX1 in a clinically relevant concentration (IC_50_ 3.93 µM; clinical plasma C_max_ 2.50–8.12 µM) [115].

#### The Role of AOX1 in Cancer

There are scarce data on the role of this enzyme in cancer. Expression of AOX1 is decreased in prostate cancer, when compared to normal prostate tissue, and is negatively correlated with Gleason score. Furthermore, its decrease was more pronounced in metastatic prostate cancer, compared to a primary tumor. This finding is suggestive of a close relationship between a gradual loss of AOX1 expression and prostate cancer occurrence and progression [116,117]. A large study in Sweden, involving 1053 men diagnosed with prostate cancer, of which 245 died from the disease, was performed to test 6 126 633 SNPs for association with prostate-cancer-specific survival. The one SNP that reached genome-wide significance (*p* < 5 × 10^−8^) and replicated in an independent cohort: rs73055188 (*p* = 5.27 × 10^−9^, per-allele hazard ratio [HR] = 2.27, 95% confidence interval [CI] 1.72–2.98) was in the intron of the *AOX1* gene. This SNP was found to be associated with prostate-cancer-specific survival time, while lower *AOX1* gene expression was correlated with shorter time to recurrence of prostate cancer [118].

In chronic pancreatitis tissue, AOX1 is strongly expressed, but it is completely absent in the malignant component of pancreatic ductal adenocarcinoma [119]. Sing et al. have reported that resveratrol prevents estrogen-mediated downregulation of AOX1 in mammary tissue and protects it against estrogen-mediated carcinogenesis [120]. In ovarian cancer, *AOX1* was shown to be one of the top downregulated genes, and *AOX1* knockdown in ovarian cancer cells, OVCAR-3 and CAOV-3, inhibited apoptosis [121]. In colorectal cancer, downregulation of AOX1 has been reported to be a consequence of *AOX1* hypermethylation [122]. In urinary bladder cancer progression, gradual AOX1 decline has been documented to be a consequence of EZH2-mediated epigenetic silencing. Downregulation of AOX1, in this type of cancer, was accompanied with an accumulation of kynurenine, as a consequence of activation of the kynurenine pathway. The mechanism by which Trp is shunted into the kynurenine pathway, upon *AOX1* silencing, has not been established and warrants further investigation [123].

While the majority of studies convincingly show that downregulation of AOX1 is related to many types of cancer, a recent study of AOX1 in colorectal cancer showed that its high expression promotes invasion, and inhibits apoptosis, via ROS production. Moreover, AOX1 upregulated expression of CD133, one of the tumor stem cell markers, at the transcriptional and protein level. On the contrary, in AOX1^−/−^APC^min/+^ mice, the expression of CD133 in cancer tissue was significantly decreased, and the survival rates were increased [124].

Although the involvement of AOX1 activity has not been addressed directly, some insight may be gained from the study aimed to explore the role of high NNMT expression in glioblastoma. In this type of tumor, NNMT compromises leucine carboxyl methyl transferase 1 (LCMT1)-mediated methylation of tumor suppressor protein phosphatase 2 (PP2A) and has been identified as a negative prognostic factor [76]. Reduction in methylation inhibits PP2A activity while it concomitantly activates oncogenic serine/threonine kinases (STKs). Apart from the expected influence of NNMT on methylation potential of LCMT1, which has been reported [66] to be sensitive to fluctuations in SAM level, the mechanism by which PPZ treatment increases methylation, and activates PP2A in glioblastoma, has not been elucidated in the quoted study [76]. Some parts of the established experimental design aimed to explore the direct effect of PPZ on PP2A methylation, in three types of U87 glioblastoma cells (WT, NNMT knockout, and NNMT overexpressing), are problematic. Namely, all U87 cell types, treated and untreated with PPZ, were concomitantly exposed to different concentrations (50, 100, and 250 nM) of PP2A inhibitor okadaic acid (known to half-maximally inhibit PP2A methylation at concentration of 40 nM [125]). Crucial information, relevant to the level of methylated PP2A in three types of U87 glioblastoma cells treated only with PPZ has not been addressed. Therefore, the extent of PPZ mediated increase in PP2A methylation, in WT and NNMT-overexpressing U87 cells, is likely highly blunted by the okadaic acid effect. Nevertheless, the presented data on PPZ-mediated reversal of NNMT-mediated inhibition of PP2A should be considered together with a recent report, which revealed PPZ as the very potent inhibitor of AOX1 [126]. Here, some critical events need to be put in a logical order: 1. AOX1 metabolizes MNAM to 2-PY and 4-PY, H_2_O_2_ is generated. 2. The activity of PP2A has been shown to be rapidly inhibited by H_2_O_2_. 3. H_2_O_2_ induces oxidation of glutathione (GSH) to oxidized glutathione (GSSG), which is also able to inhibit the activity of PP2A in a concentration dependent manner. 4. The inhibitory effect of GSSG on PP2A can be reversed by GSH, suggesting that PP2A activity can be regulated by glutathionylation [127]. It is reasonable to hypothesize that a decline in H_2_O_2_ production accompanies PPZ-mediated inhibition of AOX1, and this mechanism should be considered as a possible contributor to activation of PP2A, as it was observed, but not properly explained, in Palachinamy’s study [76].

Thioridazine, another AOX1 inhibiting drug, induces cell death and inhibits self-renewal in a variety of cancer cells. In some, but not all, triple-negative breast cancer cells, in which dopamine receptor 2 (DRD2) promotes self-renewal via a STAT3- and IL-6-dependent mechanism, thioridazine inhibits self-renewal through DRD2 inhibition. In all tested breast cancer cell lines, however, thioridazine induces G1 arrest and loss of cell viability independent of DRD2 inhibition [128]. This suggests the existence of an alternative regulatory mechanism, possibly involving AOX1, which has not been explored in the cited study [128].

The beneficial effect, of simultaneous application, of tamoxifen and cisplatin can be measured through delayed development of cisplatin resistance in human head and neck squamous-carcinoma cell lines UM-SCC-10B and UM-SCC-5 [129]. Tamoxifen was recently shown to be an AOX1-inhibiting drug [130].

Strong support to the notion that AOX1 activity is involved in cellular NAD^+^ depletion comes from an accidental finding of a large increase in NAD^+^ level in cells treated with raloxifene, an AOX1 inhibitor, while screening over 300 compounds during the development of an assay for rapid dinucleotide measurement [131]. This is, to the best of our knowledge, the only report of the effect of an AOX1 inhibitor on cellular NAD^+^ level. Considering an important role of NAD^+^ metabolism, particularly regarding the involvement of low NAD^+^ levels in promoting the acquisition of stem cell properties [63] and therapy resistance, close investigation of AOX1′s contribution to cellular NAD^+^ depletion is needed.

## 5. Concluding Remarks and Open Questions

Urgently needed development of successful therapeutic approaches asks for defining crucial nodes that govern transition between different cellular phenotypic states within a malignant tumor. This is a major challenge.

NNMT has an important role in maintaining cellular homeostasis. It methylates NAM, when its cellular level rises above the capacity of NAD^+^ salvage pathway to recycle it to NAD^+^. Thus, NNMT prevents NAM’s inhibition of NAD^+^-dependent signaling reactions, mediated by PARPs and SIRTs. Expression of NNMT is often elevated in cancer, and is associated with the acquisition of stem cell properties, metastasis, and worse clinical outcomes. The sources of critical NAM elevation in cancer cells, which would activate NNMT to the extent that would restrict the availability of SAM for other cellular methylation reactions, have not been defined. The possibility is that elevated PARP-1 activity in cancer, either due to derailed metabolism-related generation of ROS, cancer-associated inflammation, or therapy, leads to an elevated generation of NAM, a by-product of poly(ADP-ribosyl)ation, which is not accompanied by equally elevated flow through the NAD^+^-salvage pathway. Another important source of NAM is senescence-associated expression of NAD^+^ hydrolase CD38. Systematic exploration of NAM and its metabolites MNAM, 2PY, and 4PY in cancer, related to the expression of not only NNMT, but also NAMPT (the rate-limiting enzyme in NAD^+^ salvage pathway) and AOX1 (oxidizes MNAM to 2-PY and 4-PY), may provide a better insight into their function. Tissue-specific expression of AOX1 should be considered when examining its involvement in metabolizing MNAM, especially with respect to functional consequences of its metabolites in regulation of cellular NAD^+^ level. In humans, there is only one AOX isoform, expressed most abundantly in adrenal glands, adipose tissue and liver, followed by trachea, glandular epithelium of the prostate, bone, kidney, and connective tissue.

Another facet of complexity is posed by the cellular heterogeneity of malignant tumors, where cancer cells coexist with different types of stromal cells. Within a tumor, different cells do not only compete for nutrients, but they are being exposed to regulatory metabolites with profound effects on tumor progression and immune response. One such metabolite is MNAM, which has recently been found, in high grade ovarian serous carcinoma, to be elevated in T cells, despite being produced only by tumor cells and CAFs. In T cells, MNAM induces secretion of tumor-promoting cytokine tumor necrosis factor α (TNF α). Whether other metabolites, like 2-PY and 4-PY, act only in autocrine manner or affect the surrounding cells needs to be explored. Comprehensive, detailed profiling of cancer cells, and surrounding non-tumor cells at the level of transcripts, proteins, and metabolites, during cancer progression, and in response to therapy, should be performed to gain understanding of NNMT’s role in cancer and identify possible targetable components of the NAD^+^ signaling network.

As such, comprehensiveanalyses involve experiments performed on laboratory animals, and the differences in some aspects of NAD^+^ metabolism between mice and humans should be carefully considered. In mice, a commonly used model animal in cancer research, four isoforms of AOX exist (mAOX1–4), of which only mAOX2 (highly restricted to the Bowman’s gland in the nasal cavity) and mAOX3 (expressed in liver) use MNAM as a substrate [132]. This, together with the difference in efficiency to biosynthesize NAD^+^ from tryptophan, should be taken into account when using mice models in studying NAD^+^-dependent processes.

## Abbreviations

2-PY	N1-methyl-2-pyridone-5-carboxamide
4-PY	N1-methyl-4-pyridone-3-carboxamide
AADPR	Acetyl Adenosinediphosphate Ribose
ACMS	Alpha-Amino-Beta-Carboxy-Muconate Semialdehyde
ACMSD	Alpha-Amino-Beta-Carboxy-Muconate Semialdehyde Decarboxylase
ADP	Adenosine Diphosphate
ADPR	Adenosinediphosphate Ribose
Ahcy	Adenosylhomocysteinase
AMS	Aminomuconate Semialdehyde
AOX1	Aldehyde Oxidase 1
APC	Adenomatous Polyposis Coli Protein
BCL2	B-cell Lymphoma 2
Bhmt	Betaine-Homocysteine Methyltransferase
BRCA1	Breast Cancer Type 1 Susceptibility Protein
cADPR	cyclic Adenosinediphosphate Ribose
CAF	Cancer Associated Fibroblasts
CD133	Cluster of Differentiation 133
CD38	Cluster of Differentiation 38
CD44	Cluster of Differentiation 44
CD44s	CD44 standard
CD44v	CD44 variable
CDH1	E-Cadherin
CDKN2A	Cyclin-Dependent Kinase Inhibitor 2A
CSC	Cancer Stem Cells
DRD2	Dopamine Receptor 2
EGLN1	Egl Nine Homolog 1
EMT	Epithelial to Mesenchymal Transition
EMT-TFs	EMT-Inducing Transcription Factors
ESRP1	Epithelial Splicing Regulatory Protein 1
EZH1	Enhancer of Zeste 1
EZH2	Enhancer of Zeste 2
GAD3	Probable Aldehyde Oxidase gad-3
GSH	Glutathione
GSK3β	Glycogen Synthase Kinase 3β
GSSG	Oxidized Glutathione
hESC	human Embryonic Stem Cells
HGSC	High Grade Ovarian Serous Carcinoma
HIF1α	Hypoxia Inducible Factor 1 Alpha
HNCC	Head and Neck Carcinoma Cells
HNF1 β	Hepatocyte Nuclear Factor 1β
IDO	Indoleamine 2,3-dioxygenase
IL-6	Interleukin 6
iPSC	induced Pluripotent Stem Cells
LCMT1	Leucine Carboxyl Methyltransferase 1
LCMT1	Leucine Carboxyl Methyl Transferase 1
mAOX1	mouse Aldehyde Oxidase 1
MAPK	Mitogen-Activated Protein Kinase
Mat1a	Methionine Adenosyltransferase I alpha
MET	Mesenchymal to Epithelial Transition
MNAM	1-Methylnicotinamide
MOCOS	Molybdenium Cofactor Sulfurase
NA	Nicotinic acid
NAAD	Nicotinic Acid Adenine Dinucleotide
NAD^+^	Nicotinamide Adenine Dinucleotide
NADS	Nicotinamide Adenine Dinucleotide Synthetase
NAM	Nicotinamide
NAMN	Nicotinic Acid Mononucleotide
NAMPT	Nicotinamide Phosphoribosyltransferase
NAPRT	Nicotinic Acid Phosphoribosyltransferase
NMN	Nicotinamide Mononucleotide
NMNAT	Nicotinamide Mononucleotide Adenylyl-Transferase
NNMT	Nicotinamide N-Methyltransferase
NR	Nicotinamide Riboside
NRK	Nicotinamide Riboside Kinase
NRF1	NF-E2-Related Factor 1
NRF2	NF-E2-Related Factor 2
OXPHOS	Oxidative Phosphorylation
PARP	Poly [ADP-ribose] Polymerase
PHD2	Prolyl Hydroxylase 2
PI3K	Phosphatidylinositol 3-Kinase
PP2A	Protein Phosphatase 2
QPRT	Quinolinate Phosphoribosyltransferase
ROS	Reactive Oxygen Species
SAM	S-Adenosyl Methionine
SASP	Senescence-Associated Secretory Phenotype
SIRT1	NAD-Dependent Protein Deacetylase Sirtuin-1
SNP	Single Nucleotide Polymorphism
STAT3	Signal Transducer and Activator of Transcription 3
SUV39H1	Suppressor of Variegation 3-9 Homolog 1
TDO	Tryptophan 2,3-dioxygenase
TGFβ	Transforming Growth Factor-β
TIS	Therapy-Induced Senescence
TME	Tumor Microenvironment
TP53	Tumor Suppressor p53
Trp	Tryptophan
ZEB1	Zinc Finger E-box-Binding Homeobox 1
